# In memoriam: Stephen J Seligman, MD: Adverse reactions to the yellow fever vaccine: from epidemiological risk factors to causes and mechanisms

**DOI:** 10.1007/s10875-021-01204-2

**Published:** 2022-01-14

**Authors:** Jean-Laurent Casanova, Qian Zhang, Paul Bastard, Emmanuelle Jouanguy

**Affiliations:** 1 St. Giles Laboratory of Human Genetics of Infectious Diseases, Rockefeller Branch, The Rockefeller University, New York, NY USA; 2 Laboratory of Human Genetics of Infectious Diseases, Necker Branch, INSERM U1163, Necker Hospital for Sick Children, Paris, France; 3 University of Paris, Imagine Institute, Paris, France; 4 Department of Pediatrics, Necker Hospital for Sick Children, AP-HP Paris, France; 5 Howard Hughes Medical Institute, New York, NY USA

Steve J Seligman (2/4/1931–11/11/2021) has passed away at the age of 90 years. In 2011, he joined our laboratory at the Rockefeller University, New York, as an Adjunct Professor, his primary affiliation remaining New York Medical College, Valhalla, where he had been a Research Professor since 2001. Most of his career was spent at SUNY, Downstate Medical Center, Brooklyn, where he had been an Associate (1968–1981), Full Professor of Medicine (1981–2000), and then finally Professor Emeritus (2000–2021). Before that, he was a Fellow (1961–1963) and Assistant Professor in Medicine (1963–1968) at UCLA (1961–1963). He trained as a Resident at Cornell Bellevue Hospital (1956–1957), Tufts Boston City Hospital (1959–1960), and Boston Veterans Administration (1960–1961), after attending medical school at NYU (1952–1956) and college at Harvard University (1948–1952). Importantly, he also served with the Public Health Service as a USPHS Epidemic Intelligence Officer at UCLA and USC from 1957 to 1959. These two years triggered his life-long interest in the global epidemiology of infectious diseases.

During a conversation about life-threatening adverse reactions to the yellow fever (YF) vaccine, Adolfo Garcia-Sastre (Professor at Mount Sinai, New York) recommended that he contact us. In 2011, Steve wrote his first letter to us: “Until 2001, yellow fever vaccine was considered the safest of the live virus vaccines. However, cases began to be reported of reactions to the vaccine termed yellow fever vaccine-associated viscerotropic disease (YEL-AVD). Estimates of the incidence of YEL-AVD are about 0.3–0.4 per 100,000. The disease is multisystemic and resembles yellow fever caused by a wild-type virus. Viral sequences isolated from the patients have not shown any significant differences from the vaccine. Two risk groups had been identified: vaccine recipients ≥ 60 and those who had been thymectomized because of thymomas. I have recently described a third group, women of child-bearing age in whom so far the disease has invariably been fatal in contrast to the 21% case fatality rate in men ≥ 60”.

What Steve did not know is that we had already collected samples from some of these rare and tragic cases following the publication in the *Lancet* of the first papers describing severe diseases caused by the live attenuated vaccine [1, 2]. Our aim was to test the hypothesis that these patients carry inborn errors of immunity, conditions that had previously been revealed by adverse reactions to other live attenuated vaccines, including viruses [3–6]. We immediately invited him to join our laboratory and to take part in the efforts that we had begun in 2001, initially in Paris and then with our new neighbors at Rockefeller University, Peggy MacDonald and Charlie Rice, to understand the molecular basis of these dramatic adverse reactions. Despite Steve’s insistence that there were several “epidemiological groups” at risk of adverse reaction, we did not pay much attention to this aspect of his research at the time. In all honesty, we had not even read his papers, including a fascinating case of disseminated HSV-2 infection in a woman with thymic dysplasia [7], before reading his e-mail message. Moreover, in our response to him, we wrote that “these women certainly carry inborn errors of immunity,” a polite way of saying that we geneticists did not really care very much about elderly patients and those with thymoma! We now know how right he was.

In 2004, Steve first expressed his concerns regarding the safety of live flavivirus vaccines in a brief review in the *Lancet* based on the lessons learnt from the use of live attenuated YF vaccine [8, 9], which had been considered one of the safest vaccines available since 1935 [10]. Also in the *Lancet*, but one issue later, Rachel Barwick Eidex summarized the total of 23 cases of YF vaccine-associated viscerotropic disease (YEL-AVD) reported since the first known case in 1996 [11]. Four of these 23 cases were associated with thymic diseases and thymectomy [11]. Over the next decade, Steve persistently collected and studied the rare cases reported worldwide, eventually developing a systematic classification of the various risk groups for YEL-AVD [12–15]. In parallel, human genetics was entering a new era, with both next-generation sequencing and the human reference genome becoming much more widely available. After joining our laboratory, he was at last able to expand his studies of risk groups, including age, sex, and surgical history, such as thymectomy, to genomic studies. Nevertheless, we were initially reluctant to consider the patients as belonging to any particular “risk group.” After all, if one can sequence the entire genome of each individual, why does it matter which risk group they belong to [16]?

Unsurprisingly, given our “monogenic” bias [17], we started our genetic study with children. Indeed, Steve had already defined a fourth, separate risk group: fatal cases in infants and children [13, 15]. We identified in 2019 a first patient with an inborn error of type I interferon immunity: IFNAR1 deficiency [4]. Surprisingly, this 14-year-old Brazilian adolescent with life-threatening YF vaccine disease had a recessive complete deficiency of IFNAR1. She had previously been healthy and normally resistant to the other viruses to which she had been exposed. This study pointed us in the direction of type I interferons (IFN), but what about the other three groups: women in their 20 s, patients with thymoma, and the elderly?

The answer for these three risk groups emerged one year later, in 2020, when autoantibodies against type I IFNs were discovered to cause life-threatening COVID-19 pneumonia [18]. Women with systemic lupus erythematosus (SLE), men older than 65 years, and patients with thymoma (often treated by thymectomy) were known or shown to have a high frequency of autoantibodies against type I IFNs. The occurrence of auto-Abs against type I IFN in women with SLE was first reported in 1982 [19] and that in patients with thymoma was first reported in 1997 [20]. We found that the prevalence of these auto-Abs was about 0.3% until the age of 65 years, subsequently rising to 4% in those over 80 years old [21]. We were, thus, finally able to join up the dots between these conditions and YEL-AVD. A few months later, these same autoantibodies, neutralizing high concentrations of type I IFN, were identified in three patients with YEL-AVD [3] (a third of our cohort). Moreover, a fourth patient, aged 13 years, had inherited IFNAR2 deficiency [3, 5], confirming the causal relationship between inborn errors of type I IFN immunity or autoantibodies against type I IFNs and adverse reactions to the YF vaccine.

**Fig. 1:**
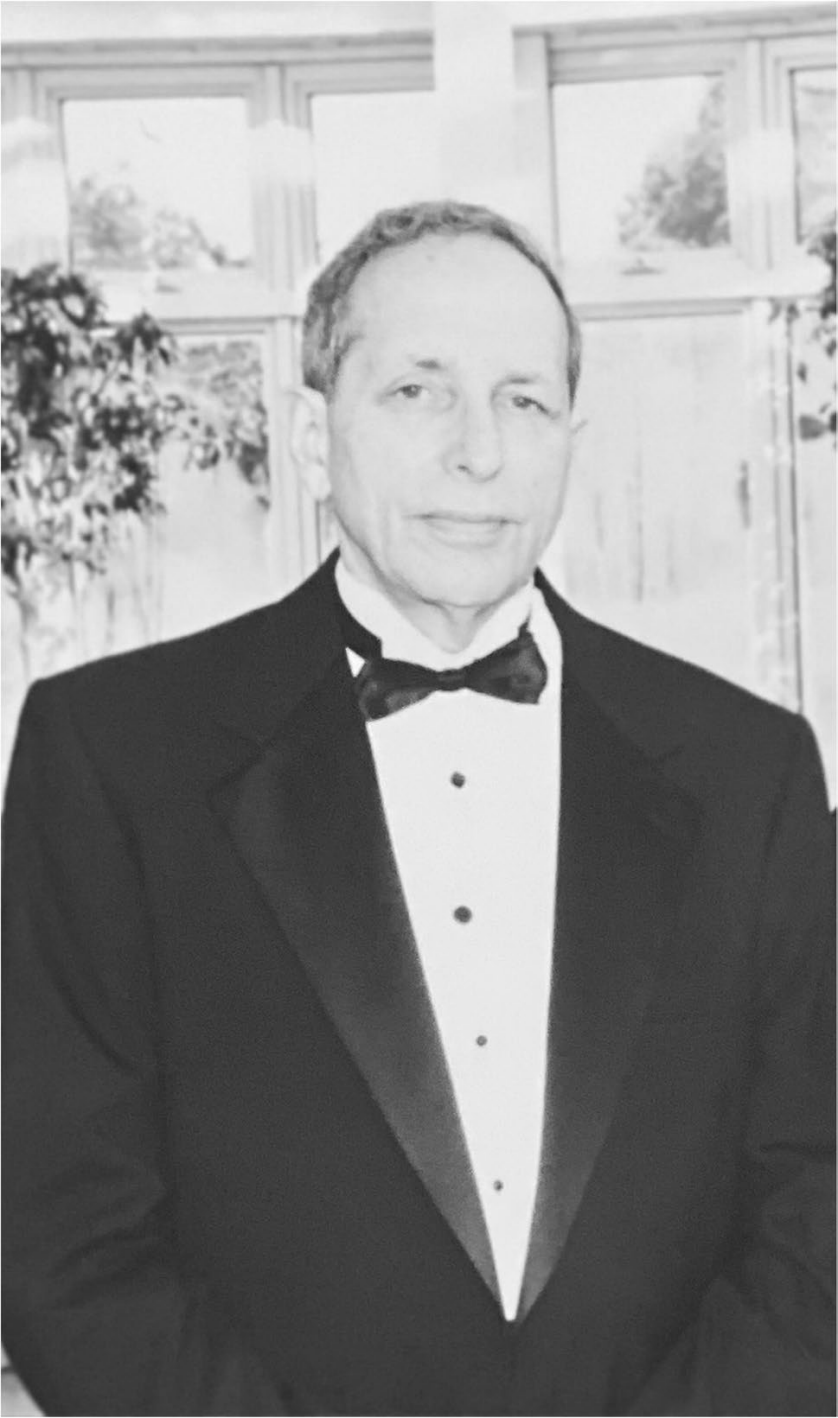
Steven J Seligman (photo courtesy of his son Marc)

Looking again at Fig. 1 in Steve’s 2014 paper in *Vaccine*, it is clear that it can serve as a road map for the human genetic and immunological discoveries we made with him years later [13]. It now seems probable that most adverse reactions to the YF vaccine are due to inborn errors of type I IFNs (especially in children and adolescents, but also in adults) or the presence of auto-Abs against type I IFNs (especially in young women, patients with thymoma, and the elderly, but also in children with other autoimmune diseases). In that light, it is troubling and inspiring that Steve had reported in 1974 the tragic but fascinating clinical course of a woman with disseminated HSV-2 infection and thymic dysplasia [7].

How could a small epidemiological study of the clinical features of only 64 patients predict so accurately the genetic and immunological determinants of adverse reactions to the YFV 17D virus? Perhaps the answer lies in Steve’s own words in 1996: “Persons who are effective in research have skills that differ from those of effective teachers and clinicians. These skills include the ability to generate research ideas, to access sufficient research resources, to obtain satisfaction from long-term results that is sufficient to continue frustrating tasks, and to persist in publishing and disseminating their results” [22]. Steve’s seminal ideas eventually bore fruit, with important implications for both the prevention and treatment of adverse reactions to YF vaccination. Commenting on this success, Steve wrote “Sometimes the most innocent can make a fruitful suggestion (smiley emoji)”.

In his last e-mail to us a few months ago, entitled “What’s happening with me,” Steve wrote that “Unfortunately, I am not in good health and am living in an independent living facility. I don’t feel up to participating in conference calls. However, I am on the lookout for reports in which there is likely to have been an underlying genetic defect and will try to prepare something for us to submit. Thanks for giving me the opportunity to participate in work with your team.” At the age of 90, right up to the end, he continued working to help us understand the root cause of infectious diseases, including adverse reactions to the YF vaccine, in particular, a field to which he had made key contributions. He also hoped to write a review summarizing his 20-year-long successful quest. This obituary is not as accurate and detailed a story of this journey as he would have written. It is of particular note that, having published his first paper in *Virology* in 1959 aged 28 years [23], it was during his last two decades, between the ages of 70 and 90, that he made the most important scientific contributions of his career, demonstrating that intelligence and enthusiasm do not necessarily decline with age. The energy and passion that emanated from him during our weekly laboratory meetings was an inspiration to everyone, but particularly for those embarking on their research careers.

Steve was also a great father, grandfather, and husband. The kindest of men. A scientist, physician, and teacher who gave his all to trying to help sick people get better. He also had three core values at heart. The first was respect for science. He was dismayed at the dismissiveness of so many regarding the incredible scientific research performed of late, which has saved countless lives. The second was respect for civil rights. He was always concerned that everyone should be treated equally in every respect and fought for this in every way possible. And, the third was respect for the planet. There is so much that we can accomplish through our own actions as people to ensure that future generations have the opportunity to enjoy Earth.

Steve will be sorely missed in our laboratory and by the biomedical community at large. He is survived by his sons, William and Marc Seligman, grandchildren Marisa and Evan Seligman, and his wife Hannelore Seligman, together with her children and their families: Andrew Mackles and his spouse Kristen Petterson-Mackles, their children Jeremy and Benjiman Mackles, and Paul and Ronnie Mackles, and their son Trevor Mackles.
